# Supplementary material for: Venomix: a simple bioinformatic pipeline for identifying and characterizing toxin gene candidates from transcriptomic data
Source: PeerJ. 2018 Jul 31;6:e5361. doi: 10.7717/peerj.5361 (PMC6074769; doi:10.7717/peerj.5361)
Supplement: Supplemental Information 3 [file peerj-06-5361-s003.gz › FinalOutput_E-6/Thrombin-like_enzyme_TLBan_1/finaltree.pdf]

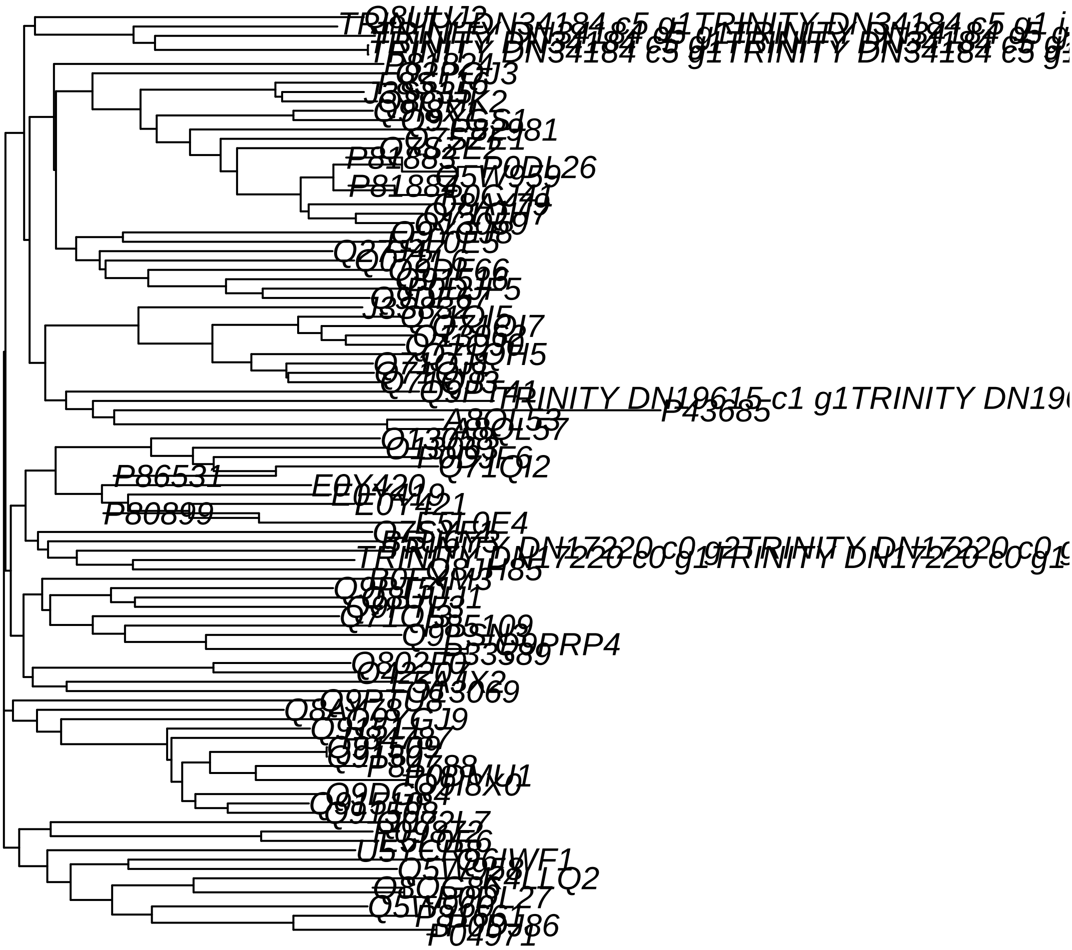
P43685
P86531
P80899
E0Y420
Q71QI2
Q8QL57
Q5W959
Q9DGJ5
Q42583
P04971
